# SMART: structured, meaningful, auditable, responsible, and transparent documentation for clinical AI

**DOI:** 10.1093/jamia/ocag117

**Published:** 2026-08-19

**Authors:** Ankur Lohachab, Frédéric Jung, Stef Rommes, Gökhan Ertaylan, Anshu Ankolekar, Visara Urovi

**Affiliations:** Institute of Data Science, Department of Advanced Computing Sciences, Maastricht University, Maastricht, Limburg, 6229 GS, The Netherlands; Environmental Intelligence, Vlaamse Instelling voor Technologisch Onderzoek (VITO), 2400 Mol, Antwerp, Belgium; Environmental Intelligence, Vlaamse Instelling voor Technologisch Onderzoek (VITO), 2400 Mol, Antwerp, Belgium; Environmental Intelligence, Vlaamse Instelling voor Technologisch Onderzoek (VITO), 2400 Mol, Antwerp, Belgium; D-Lab, Department of Precision Medicine, GROW - Research Institute for Oncology and Reproduction, Maastricht University, Maastricht, Limburg, 6229 ER, The Netherlands; Maastro Clinic, Maastricht, Limburg, 6229 ET, The Netherlands; Institute of Data Science, Department of Advanced Computing Sciences, Maastricht University, Maastricht, Limburg, 6229 GS, The Netherlands

**Keywords:** blockchain, medical records systems computerized, artificial intelligence, documentation, reproducibility of results, electronic health records

## Abstract

**Objective:**

Clinical AI systems and models increasingly need traceable documentation that makes intended use, performance evidence, transparency, and lifecycle status explicit, yet existing model cards, datasheets, reporting guidelines, and other documentation approaches do not consistently bring together structured clinical fields, vocabulary-linked cohort descriptions, structured performance reporting, and governed documentation history. We introduce Structured, Meaningful, Auditable, Responsible, and Transparent (SMART), a framework that adapts the model card concept into structured, lifecycle-aware documentation with OMOP integration, role-based lifecycle governance, and a blockchain-backed audit trail for verifying documentation integrity and lifecycle history.

**Materials and Methods:**

Following a design-science-informed approach, we analyzed documentation requirements, reviewed existing approaches, implemented SMART through open-source packages and smart contracts, and evaluated it using example SMART model cards, an illustrative COPD exacerbation risk-prediction documentation case study, and blockchain-governed lifecycle experiments.

**Results:**

The design process resulted in the SMART framework in which documentation provisions are reflected across Schema, Lifecycle, and Chain (ie, Blockchain) components. Evaluation showed that, in multi-party settings, documentation history remains independently verifiable because lifecycle actions are role-attributed and linked to content hashes; median testnet gas use ranged from 352 722 to 852 399 gas across the evaluated lifecycle operations.

**Discussion:**

SMART positions clinical AI documentation as a shared infrastructure for preliminary model assessment, making role attribution, lifecycle state, and documentation changes more transparent and traceable over time.

**Conclusion:**

By providing an implementable framework, SMART illustrates how clinical AI documentation can be structured, clinically contextualized, auditable, responsible, transparent, and lifecycle-aware.

## Introduction

AI systems and models used in clinical settings for tasks such as image interpretation, risk stratification, and treatment planning^1^^,^^2^ can fall within the scope of multiple regulatory frameworks and guidance documents, depending on their intended purpose and context of use. In the European context, for example, software intended by the manufacturer for diagnostic or therapeutic purposes may qualify as a medical device under the EU MDR,^3^ with expectations related to clinical evaluation, post-market surveillance, device traceability, and General Safety and Performance Requirements. Beyond medical-device qualification, the EU AI Act^4^ introduces AI-specific requirements by classifying certain AI systems used as, or as safety components of, medical devices as high-risk when the device is subject to third-party conformity assessment, with associated expectations for technical documentation, data governance, transparency, and post-market monitoring. Outside the European Union, FDA guidance in the United States^5^^,^^6^ discusses lifecycle management for AI-enabled device software functions, including predetermined change control plans for planned modifications. In this context, we argue that traceable documentation is important for reducing compliance uncertainty, supporting accountability, and enhancing transparency in documentation when clinical AI models or software-based systems are deployed. It can be noted that our interpretation of the regulatory and guidance documents mentioned here is intended to motivate documentation needs rather than to provide legal interpretation or compliance advice, and, as these regulations and guidance continue to evolve, the specific requirements we reference may be subject to further adjustment.

Nevertheless, many existing documentation approaches are not yet designed to consistently document known risks in clinical AI. Such risks include performance disparities across demographic subgroups^7^^,^^8^ and degradation across institutions,^9^ which may remain difficult to assess when documentation does not clearly distinguish developer-reported performance from other evaluated performance evidence. For instance, the Epic Sepsis Model achieved an AUC (ie, AUROC) of 0.63 in independent validation vs developer-reported AUCs of 0.76-0.83,^10^ illustrating how performance claims and validation results can diverge. More broadly, in a survey of 43 US health systems, only 38% of clinical risk stratification adopters reported high success,^11^ suggesting that institutions need better tools to assess evidence before committing to local adoption. While several approaches have addressed model documentation more broadly, they have generally focused on different parts of the documentation problem. For example, model cards^12^ and FactSheets^13^ provide templates for recording model characteristics and intended use, and datasheets for datasets^14^ standardize training data reporting; all 3 are general-purpose rather than clinical-specific. More recently, Gilbert et al.^15^ argued that health AI model cards should integrate with regulatory labeling and link to verified, auditable information. However, to the best of our knowledge, existing approaches do not yet bring together structured clinical-context fields, vocabulary-linked cohort descriptions, structured performance reporting, and a governed lifecycle in which published documentation is immutable, can be withdrawn through deprecation, and can be superseded by linked successor versions within a single framework.

To this end, we present Structured, Meaningful, Auditable, Responsible, and Transparent (SMART), a framework for structured clinical AI documentation.^16^ SMART adapts the model card concept^12^ for clinical AI through 4 integrated building blocks: creation of SMART model cards for structured clinical context and performance reporting; OMOP CDM integration for vocabulary-linked cohort descriptions^17^; a role-based lifecycle for review, publication, withdrawal, and version lineage; and a blockchain audit trail for verifiable documentation history.^18^ The SMART model card creation and OMOP integration capabilities are implemented as Python packages (smart-model-card^19^^,^^20^ and smart-omop^21^); the lifecycle and audit mechanism are implemented as smart contracts, first tested on a local Hardhat network and then evaluated on the Polygon Amoy testnet.

## Methods

This study follows a design-science-informed approach,^22^ summarized in Figure 1: we analyzed documentation requirements, reviewed existing approaches, implemented SMART, and evaluated it using example SMART model cards, an illustrative COPD exacerbation risk-prediction documentation case study, and blockchain-governed lifecycle experiments.

**Figure 1.**
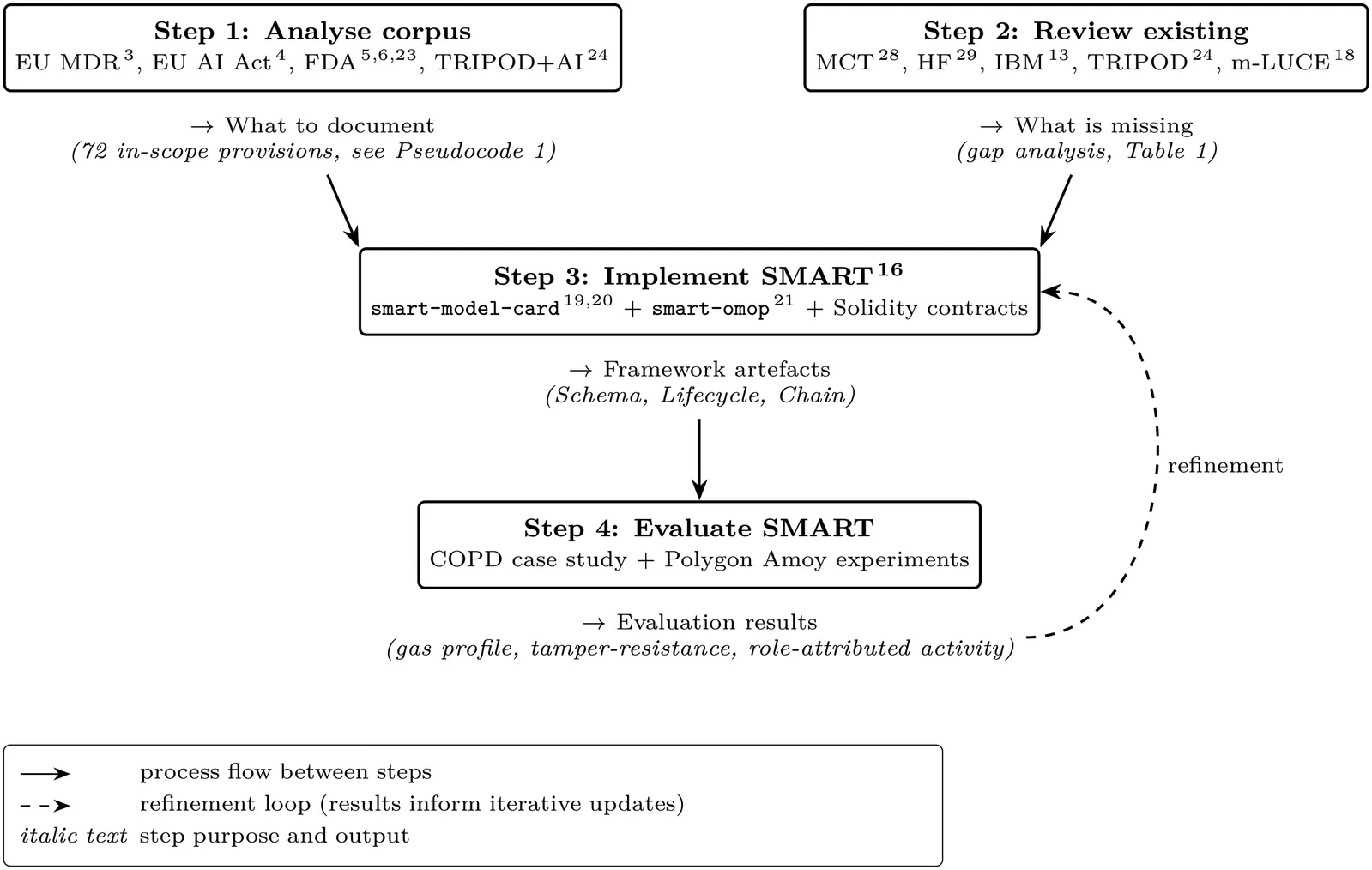
Overview of the workflow followed in this study.

Pseudocode 1 Regulatory-provision extraction and SMART component attribution.

**Inputs:** 

*D*: source PDFs (EU MDR, EU AI Act, 3 FDA documents, TRIPOD+AI)

V=〈B,Π,A,Σ,C〉 : bearer vocabulary *B*, predicate vocabulary Π, artifact vocabulary *A*, synonym table Σ, and artifact-to-component map C

$E=\{e_{\mathrm{rgx}},e_{\mathrm{qwen}},e_{\mathrm{llama}}\}$ : a rule-based (regex) extractor and 2 70B-class LLM extractors

K={Schema,Lifecycle,Schema+Lifecycle,Lifecycle+Chain, Schema+Lifecycle+Chain,Chain} : in-scope SMART components

**Output:** 

*S*: in-scope provisions represented by SMART components, with S⊆F⊆Q. Unresolved classes (Q∖F) and resolved but out-of-scope classes (F∖S) are retained as diagnostics.

1. Ingest.   T←{t∣t∈paragraphs(d), d∈D, t contains a deontic modal}

 ▹  *Paragraphs are split on blank lines, retained with document and locator metadata, and filtered by modal terms.*

2. Extract.   $R\leftarrow \underset{t\in T,\;e\in E}{⊎}e.\text{extract}(t)$

 ▹  *Each extraction is a raw* $\left(b_{\text{raw}},\pi_{\text{raw}},a_{\text{raw}}\right)$  *triple anchored to a verbatim text span.*

3. Canonicalize.   Q←canonicalize(R,V)

 ▹  *Synonym lookup maps each slot to B,* Π, or A; unmatched slots are marked Unknown_*. Equivalent triples are grouped as canonical classes.

4. Resolve.   F←{q∈Q∣q has no Unknown_* slot}

 ▹  *Classes with unresolved vocabulary terms are retained for later curation.*

5. Attribute.   S←{f∈F∣C(f.a)∈K}

 ▹  *Resolved provisions are kept when their artifact maps to an in-scope SMART component.*

**Outcome on this corpus:**  |T|=420, |R|=10,921, |Q|=183, |F|=98, |S|=72; |Q∖F|=85 vocabulary-gap classes and |F∖S|=26 resolved but out-of-scope classes.

First, we analyzed documentation and governance provisions in a source corpus of public regulatory, guidance, and reporting documents. The corpus included medical-device regulation (EU MDR^3^), AI-specific regulation (the EU AI Act^4^), 3 FDA documents addressing AI/ML-enabled device software and lifecycle change management (an AI/ML SaMD action plan,^5^ draft lifecycle-management guidance,^6^ and predetermined-change-control guidance^23^), and clinical-prediction reporting guidance (TRIPOD+AI^24^). These documents were split on blank lines and filtered to paragraphs containing at least one deontic modal term (eg, *shall*, *must*, *should*, *may*), yielding 420 paragraphs: 164 from the EU MDR, 137 from the EU AI Act, 105 from the 3 FDA documents, and 14 from TRIPOD+AI. In simple terms, deontic modals express obligation, permission, or recommendation, and characterize prescriptive language in regulatory and guidance documents^25^. These paragraphs were then processed as summarized in Pseudocode 1. Note that locator metadata in the extraction outputs uses the PDF page index from the parsed file. Page references in the manuscript use the source documents’ own page numbering where available; for some documents this matches the PDF page index, whereas for others it differs because of front matter or cover pages. Extraction was performed using a rule-based (regex) extractor and 2 70B-class language-model extractors (Qwen-2.5-72B and Llama-3.3-70B), yielding 10 921 retained raw provision tuples: 543 from regex, 3685 from Qwen, and 6693 from Llama. Each retained tuple included a verbatim source span. The 2 language-model extractors produced 14 006 candidate tuples in total: the 10 378 Qwen and Llama tuples above were retained, and the remaining 3628 were dropped before canonicalization, of which 1091 carried a non-deontic modal and 2537 lacked a recoverable verbatim span or a complete actor, action, and artifact triple. Against a 169-item adjudicated reference set of actor, action, and artifact triples, Qwen-2.5-72B reached precision 0.70 and recall 0.88, while Llama-3.3-70B reached precision 0.62 and recall 0.79. Each retained tuple represented a responsible actor, action, and associated documentation or governance artifact. Similar provisions expressed differently across sources were normalized against a fixed closed vocabulary with synonym tables, collapsing the retained tuples into 183 canonical equivalence classes. Of these, 98 were fully resolved, meaning that every actor, action, and artifact slot mapped to a known vocabulary term. Each fully resolved provision was then attributed to SMART components using the curated artifact-to-component lookup. Of the 98 fully resolved provisions, 72 were within SMART’s design scope and spanned 16 distinct artifacts: 51 represented by Schema alone, 14 by Lifecycle alone, 6 by Schema+Lifecycle, and 1 by Lifecycle+Chain; the remaining 2 in-scope combinations, Schema+Lifecycle+Chain and Chain, received no provisions on this corpus.

Second, we reviewed existing documentation approaches, including model cards,^12^ datasheets for datasets,^14^ FactSheets,^13^ clinical-AI reporting guidance and assurance frameworks,^24^^,^^26^ health-AI model card proposals,^15^^,^^27^ and blockchain-based model card accountability work.^18^  Table 1 contrasts a selection of these approaches with SMART across a set of features. Overall, the source-corpus extraction and the review suggested that existing approaches address different aspects of clinical AI documentation, but do not yet bring together structured clinical context, standardized cohort description, structured performance reporting, benefit-risk documentation, and governed versioning in an integrated clinical-AI documentation workflow.

**Table 1.** Feature comparison of documentation approaches for clinical AI.

Feature	MCT	HF	IBM	TRIPOD	m-LUCE	SMART
Structured clinical-context fields	○	○	○	◐	○	●
OMOP CDM and vocabulary integration	○	○	○	N/A	○	●
Machine-readable cohort definitions	○	○	○	◐	○	●
Programmatic validation	◐	◐	◐	N/A	◐	●
Structured performance reporting (claimed and validated)	○	○	○	◐	○	●
Benefit-risk and surveillance-planning fields	○	○	○	◐	○	◐
Subgroup performance documentation	○	○	◐	◐	◐	◐
Device and risk classification metadata	○	○	○	N/A	○	◐
Content integrity hashing	○	◐	○	N/A	◐	●
Blockchain audit trail	○	○	○	N/A	●	●
Governed lifecycle with Role-based access control	○	○	◐	N/A	●	●
Version comparison and lineage support	○	◐	○	N/A	◐	◐

● = present; ◐ = partial; ○ = not present; N/A = outside intended scope. MCT = Google Model Card Toolkit^28^; HF = Hugging Face model cards^29^; IBM = FactSheets^13^; TRIPOD = TRIPOD+AI^24^; m-LUCE = blockchain-based model card accountability framework.^18^ Partial indicates guidance, free-text support, metadata, repository history, lifecycle-state validation, or non-enforced support rather than a structured and enforced clinical-AI documentation workflow.

Third, we implemented SMART model card creation and OMOP integration as 2 open-source Python packages: smart-model-card (v3.0.0)^19^^,^^20^ and smart-omop (v2.0.3).^21^ Briefly, the smart-model-card package provides features such as programmatic creation of SMART model cards and different ways of exporting a structurally validated card. The smart-omop package provides OMOP-oriented functionality for concept-set management, cohort retrieval, and Heracles characterization queries. To support governance and auditability, we coupled both packages to a governed lifecycle and audit mechanism implemented as Solidity smart contracts^16^ for role-attributed registration, review, publication, deprecation, soulbound token issuance, relayed transactions, and lineage-based versioning. The contracts were tested locally and evaluated on the Polygon Amoy public testnet in the lifecycle, gas-cost, and tamper-resistance experiments. In the SMART framework, on-chain records provide file-level integrity and lifecycle provenance through stored content hashes and immutable on-chain audit entries, referred to as ActionRecords, while field-level comparison is provided by the smart-model-card package.

Finally, we evaluated SMART using example model cards and blockchain-governed lifecycle experiments. We describe the SMART model card template through a clinical documentation case study for a COPD exacerbation risk predictor populated with illustrative example values. In addition, because rejection and deprecation are terminal states, the lifecycle evaluation used dedicated model card instances to cover review, revision, publication, withdrawal/deprecation, and lineage-based successor creation. At the blockchain level, the experiments assessed role-based access control, auditability, backend-independent verification from on-chain records, gas use, and successor-version linkage through a supersedes pointer.

### SMART model card template

The proposed SMART model card template (Figure 2) adapts the model card concept^12^ while considering the in-scope provisions extracted through the source-corpus analysis and the features observed across the reviewed documentation approaches (Table 1). The resulting template provides structured fields for clinical context and intended-use boundaries, OMOP-linked cohort description, structured performance reporting, and benefit-risk, safe-use, and surveillance-planning information. For example, Figure 3 shows one example SMART model card used in the evaluation, the COPD Exacerbation Risk Predictor (can be viewed on Polygon Amoy as token #641), a 12-month exacerbation risk model populated using a de-identified EHR cohort harmonized to the OMOP CDM and scoped to adults aged 40 or older with confirmed COPD diagnosis (OMOP concept 255573 and descendants; labeled in the example card as chronic obstructive lung disease) in a hospital outpatient setting.

**Figure 2.**
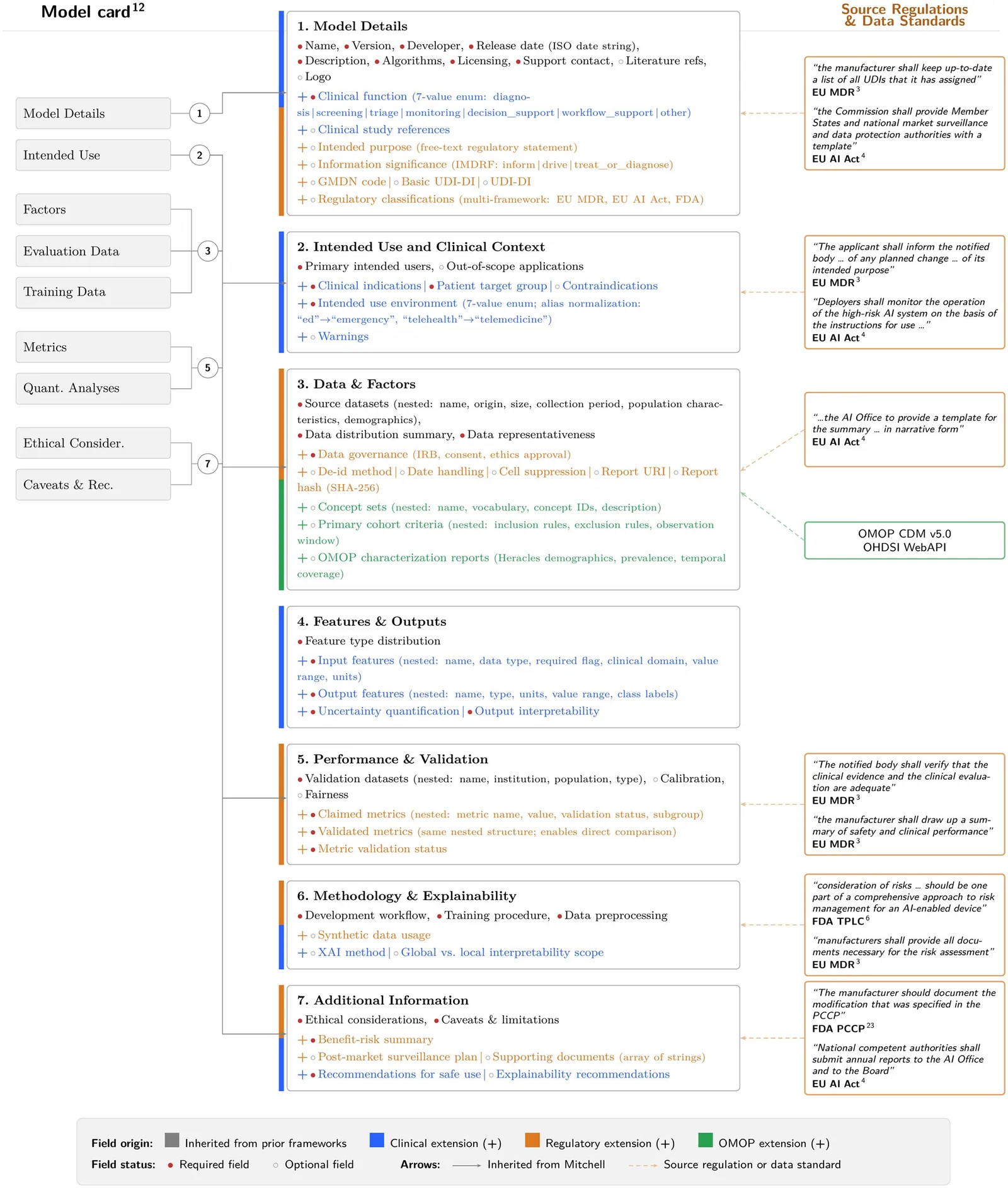
The SMART model card template adapts model cards^12^ (left) into 7 sections with clinical and OMOP extensions (center), alongside representative source excerpts (right). Circled numbers indicate the corresponding SMART section.

**Figure 3.**
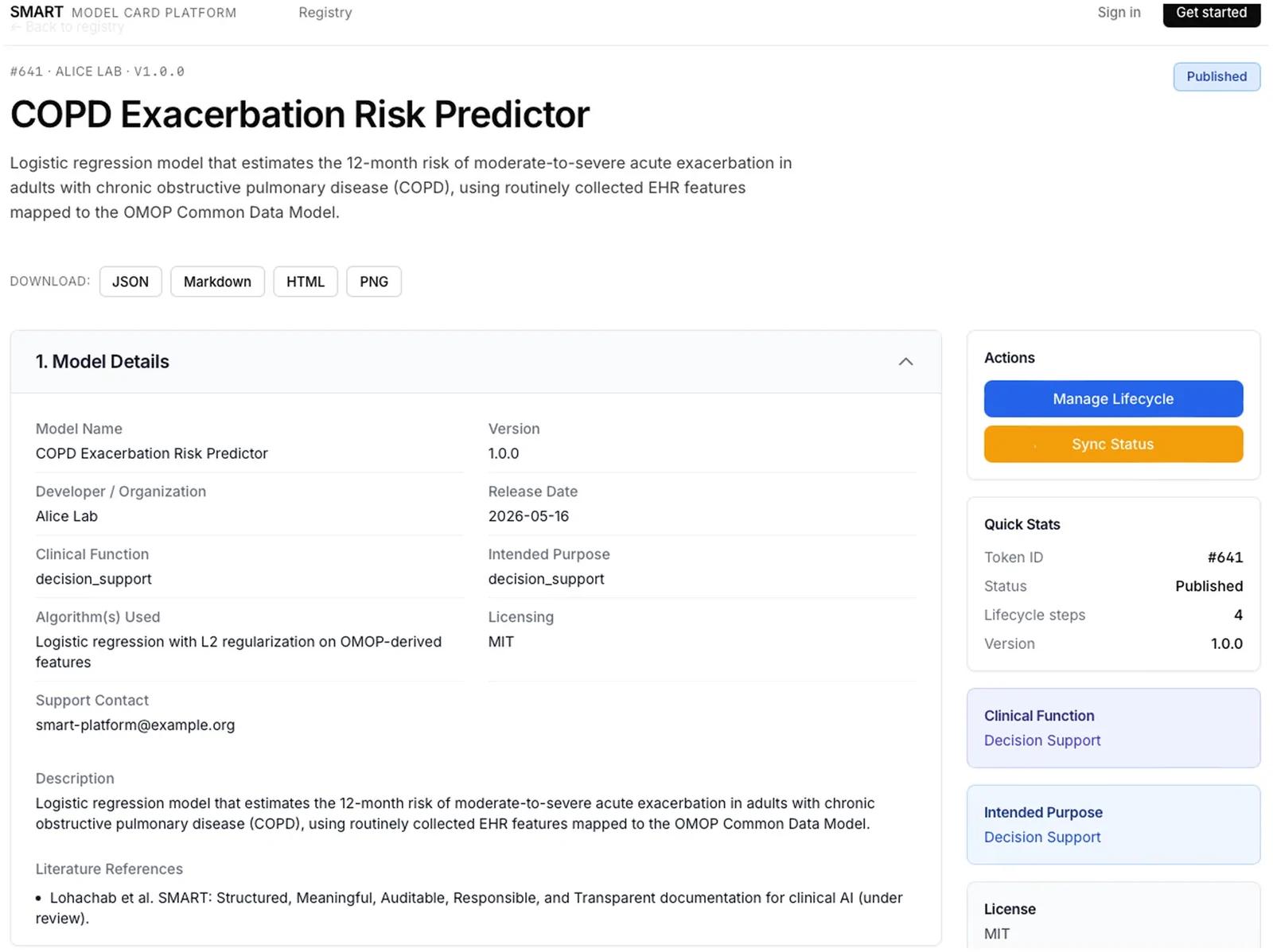
Illustrative published COPD Exacerbation Risk Predictor model card in the SMART platform (a reference implementation of the proposed framework), showing required Section 1 model-detail fields.

#### Clinical context (Sections 1-2)

Sections 1 and 2 record selected model details and intended-use context, including clinical function, intended purpose, patient target group, intended use environment, contraindications, and warnings. Rather than leaving intended use only as free text, these fields make the intended setting, users, and exclusions visible in the card. This design choice reflects the source-corpus analysis; for example, the EU AI Act states that *Deployers shall monitor the operation of the high-risk AI system on the basis of the instructions for use …* (p. 68).^4^ In the COPD Exacerbation Risk Predictor example (Figure 3), Section 1 records both the clinical function and intended purpose as decision_support, with the card description framing the model as a 12-month COPD exacerbation risk predictor. Section 2 scopes the card to adults aged 40 or older with confirmed COPD diagnosis using OMOP concept 255573 and descendants, at least 12 months of observation history, and a hospital_outpatient intended use environment. Contraindications and out-of-scope applications clarify that the model is not intended for COPD diagnosis, asthma differential diagnosis, or severity grading, and exclude pediatric patients, patients without confirmed COPD diagnosis, and patients with insufficient EHR history. The warnings state that the output is a risk estimate, not a diagnosis, and should be interpreted with clinical judgment before care decisions.

#### Data governance and vocabulary interoperability (Section 3)

Section 3 (Data & Factors) specifies source datasets, data distribution summary, data representativeness, data governance, OMOP concept sets, and primary cohort criteria. For example, Figure 2 includes the EU AI Act excerpt *…the AI Office to provide a template for the summary … in narrative form* (p. 28).^4^ In the example card, the source datasets field records an illustrative institutional OMOP CDM extract of de-identified EHR data (n=12,438; 2018-2023). The OMOP concept sets include COPD (concept 255573 and descendants), and the primary cohort criteria define adults aged 40 or older with at least one occurrence of concept 255573 and 12 months of prior observation. The data distribution summary records a 70/15/15 train, validation, and test split, and data representativeness is described as a single-network cohort. The optional de-identification and OMOP detailed-reports fields are left empty.

#### Features and outputs (Section 4)

Section 4 (Features & Outputs) specifies input features, output features, feature type distribution, uncertainty quantification, and output interpretability. For instance, in the example card, the feature type distribution summarizes 12 features (5 numeric and 7 categorical), and the output feature is a calibrated probability of moderate-to-severe exacerbation within 12 months. The uncertainty quantification field records bootstrapped 95% confidence intervals, and the output interpretability field describes per-prediction contributions based on standardized logistic-regression coefficients.

#### Performance and validation (Section 5)

Section 5 (Performance & Validation) distinguishes developer-reported metrics from evaluated metrics, allowing claimed performance and evaluation evidence to be documented side by side. For example, the EU MDR states that *The notified body shall verify that the clinical evidence and the clinical evaluation are adequate …* (p. 150).^3^ In the example card, the validation-datasets field records a held-out validation dataset, while 4 claimed metrics are reported with status Claimed (AUROC 0.78, AUPRC 0.42, Brier score 0.14, and expected calibration error 0.03); the validated-metrics field is marked pending, holding placeholder values rather than evaluated results in this example card.

#### Methodology and explainability (Section 6)

Section 6 (Methodology & Explainability) describes how the model was developed, including development workflow, training procedure, data preprocessing, synthetic data usage, explainable AI method, and global vs local interpretability scope. This is particularly relevant for communicating how model outputs should be understood; for example, the FDA lifecycle draft guidance states that *consideration of risks related to understanding information should be one part of a comprehensive …* (p. 17).^6^ For instance, in the example card, the development workflow outlines a pipeline from cohort definition via OMOP concept sets to model training and validation using L2-regularized logistic regression. The training procedure and data preprocessing fields record cross-validation, class-imbalance handling, and feature preprocessing, while the explainability fields describe standardized coefficients and per-prediction contribution decomposition.

#### Benefit-risk and surveillance (Section 7)

Section 7 (Additional Information) documents benefit-risk summary, caveats & limitations, recommendations for safe use, post-market surveillance plan, explainability recommendations, and supporting documents. In simple terms, this captures post-deployment considerations such as risk, monitoring, and safe use; for example, the FDA PCCP guidance states that *The manufacturer should document the modification that was specified in the PCCP …* (p. 40).^23^ For instance, in the example card, the benefit-risk summary notes proactive identification of high-risk patients alongside risks of over-reliance, automation bias, and dataset shift. The caveats & limitations record single-network training data, OMOP-mapped EHR dependence, absence of external validation, and possible drift with care-pathway changes. The post-market surveillance plan specifies quarterly review of calibration and subgroup AUROC, with revalidation triggered by prevalence shift greater than 10% or AUROC below 0.72.

### Governed lifecycle

The SMART model card sections capture documentation content but not, by themselves, its change history or governance. In the source-corpus extraction used to inform SMART’s design scope, lifecycle-related artifacts, mainly associated with post-market surveillance and predetermined change control, represented 21 of the 72 in-scope canonical provisions, spanning artifacts such as ChangeControlPlan, ChangeNotification, PostMarketMonitoringReport, PostMarketSurveillancePlan, and CorrectiveAction. SMART therefore includes a role-based documentation lifecycle, implemented through smart contracts. (Roles such as AIDeveloper, Reviewer, and Publisher are representative lifecycle responsibilities in the SMART implementation rather than institutional job titles.) This lifecycle governs how each SMART model card progresses from creation through review, publication, withdrawal/deprecation, and version lineage (Figure 4). Lifecycle transitions are recorded as ActionRecords on-chain, and each card is represented as a nontransferable SMART model card token bound to its creator address.

**Figure 4.**
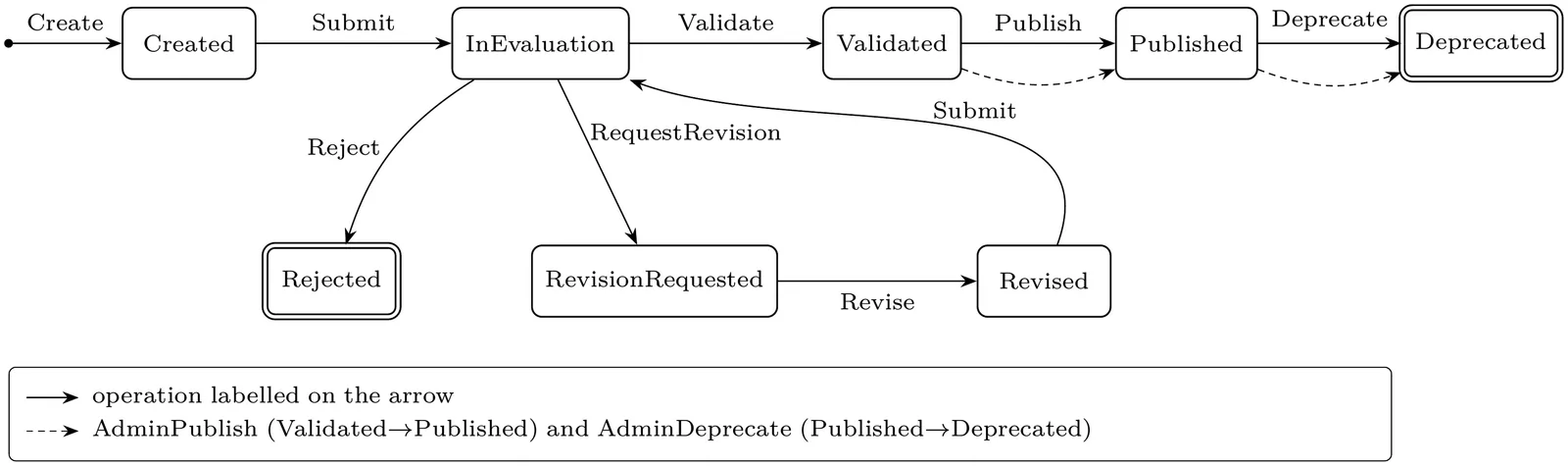
Lifecycle of SMART model cards. Doubled borders mark terminal states (Rejected: failed review; Deprecated: withdrawn after publication).

#### Role-based access control

To support lifecycle governance, the smart contracts restrict state-changing operations to designated roles. Three roles, designated in SMART as AIDeveloper, Reviewer, and Publisher, separate responsibilities at the function level; when assigned to distinct accounts, this prevents the same address from both creating and approving a model card. The AIDeveloper creates SMART model cards, submits them for review using submitForEvaluation, revises cards when changes are requested using reviseModelCard, and resubmits revised cards using submitRevision. The Reviewer reviews submitted cards and may validate them, reject them, or request revisions. The Publisher authorizes publication of validated cards and may later withdraw/deprecate published cards. This separation assigns documentation creation, review, and publication to distinct responsibilities, without implying that SMART replaces regulatory or institutional review. Admin and Reader roles support designated administrative lifecycle actions and application-level read access, respectively; on-chain records remain publicly readable by design.

#### Terminal states

Of the states described above, 2 are terminal and inactivating (double-bordered in Figure 4). Rejection is terminal: a card that fails review must be replaced by a new record starting at Created, preserving the history of unsuccessful submissions. (Example terminal rejection on Polygon Amoy: token #1 (Rejected).) Withdrawal/deprecation is likewise terminal: a deprecated card cannot be reactivated. (Example terminal deprecation on Polygon Amoy: token #641 (Deprecated); this is the same card shown as Published in Figure 3, deprecated here for the lineage experiment.) These constraints preserve 2 lifecycle invariants: published cards must first reach Validated status, including under emergency publication, and terminal records cannot be used to bypass review by reentering the lifecycle.

#### Versioning and version lineage

Since rejection is terminal, incomplete submissions that can be corrected should enter the revision loop rather than be rejected. The lifecycle, therefore, distinguishes between prepublication revision and lineage-based successor versions. For cards that need improvement during review, the revision loop provides a structured path: the Reviewer requests specific changes, the AIDeveloper revises the card, and the revised card is resubmitted for review. These revisions remain within the same SMART model card record, with up to 10 revision cycles permitted per record. (Example revision-loop trace on Polygon Amoy: token #626 includes a requestRevision followed by reviseModelCard [revisionNumber=1].) When changes are more substantial, such as a new algorithm, expanded feature set, or new validation data, SMART represents the change as a successor version with its own lifecycle. For successor versions, the SMART platform workflow requires the predecessor to be deprecated before a new linked record is created within the same version lineage. (Example successor lineage on Polygon Amoy: token #641 (v1.0.0) completed its lifecycle and was deprecated on-chain; token #642 (v2.0.0) was minted as its successor via createModelCardWithLineage, with linkage recorded in the VersionMinted(tokenId=642, supersedes=641) event, and subsequently published.)

### Blockchain audit trail mechanism

To make documentation history and lifecycle actions independently verifiable when the documentation maintainer, infrastructure operator, and relying party are distinct, SMART records lifecycle actions on a public blockchain. For instance, in conventional systems, administrative access to application, storage, and logs allows audit-trail deletion or retroactive modification (eg, a SQL operation such as DELETE FROM audit_log). In SMART, the lifecycle audit trail is append-only and cannot be removed or modified once written, while current-state fields evolve only through contract-enforced transitions that append new ActionRecords, each mirrored by a BoundAction event. Unauthorized lifecycle operations revert under role-based access control without producing forged ActionRecords. SMART further incorporates and evaluates safeguards against blockchain-specific adversarial scenarios (Table 2).

**Table 2.** Scenario-based audit-trail assessment comparing SMART with an operator-controlled reference scenario.

	SMART		Operator-controlled reference scenario	
Scenario	Prevented	Audit trail preserved	Prevented	Audit trail preserved
Audit-trail deletion	✓	✓	✗	✗
Retroactive claim modification	✓	✓	✗	✗
Forged withdrawal/deprecation	✓	✓	✗	✗
Privileged operator-as-adversary	4/5	5/5 detectable	N/A	N/A
Backend-independent verification	✓		✗	

The operator-controlled reference scenario assumes that the application, database, logs, and backend are maintained by the same operator. N/A indicates no corresponding on-chain role, soulbound-token, or public-event abstraction. “4/5” indicates that 4 of 5 privileged-operator attacks were cryptographically blocked on-chain; the fifth (role-grant followed by a forged action) succeeded but emitted public RoleGranted/RoleRevoked events, making it forensically detectable by any third party reading the contract’s events. Backend-independent verification refers to read-only verification through public RPC without using the SMART backend.

For example, attempts to transfer or re-assign the SMART model card token revert at the contract level because the token is nontransferable by design (a soulbound NFT under EIP-5192), and lifecycle calls from accounts without the required role revert under contract access-control checks. Even when a privileged role is granted to an additional account, the grant and any resulting action are emitted as public events on-chain, so role changes and their downstream actions remain externally observable. To balance storage and cost while preserving security, SMART records a SHA-256 hash of each model card JSON together with lifecycle action metadata on a public blockchain, allowing relying parties to verify whether a retrieved model card matches the version recorded at a given lifecycle action.^18^  Table 2 summarizes a representative scenario-based assessment, including backend-independent verification (read-only verification through public RPC without using the SMART backend), conducted as a trust-boundary analysis for external verifiability.

#### Content integrity and externally verifiable history

The on-chain record for each model card includes the creator address, metadata URI, SHA-256 content hash, lifecycle status, timestamps, and last modifier address; this keeps on-chain storage proportional to lifecycle actions rather than documentation size. (For example, on the mint transaction of token #641 (*COPD Exacerbation Risk Predictor*, v1.0.0), the ModelCardCreated event records creator address 0x5a78…1bb2 (developer alice.org), the metadata URI for the v1.0.0 metadata JSON in object storage, and the SHA-256 content hash 0x43a94e0c…8681c1; the same hash is re-anchored in the genesis ActionRecord (actionId=1379, transitionType=Create), mirrored by its BoundAction event.) At each lifecycle transition, the contract records the previous and new status, the actor that triggered the transition, and the associated content hash, while the backend projects the same lifecycle information into Neo4j for status lookup, lineage traversal, and search. Because the SMART model card token is nontransferable, lifecycle actions remain attributable to the addresses that performed them. To verify documentation integrity, a relying party retrieves the model card JSON from the recorded metadata URI, recomputes its SHA-256 hash, and compares it with the corresponding on-chain hash. For the currently registered SMART model card version, this hash is read from the lifecycle contract’s modelCards(tokenId) view (the ModelCard struct’s contentHash field). The change sequence is reconstructed from the per-transition ActionRecords: the genesis ActionRecord (transitionType=Create) carries the initial content hash, mirrored by a BoundAction event, and each revision records the previous hash as previousContentHash in a RevisionHistoryRecorded event. In the evaluation, this verification path reconstructed 4 lifecycle actions across 3 actors directly from on-chain reads and confirmed consistency with the lifecycle state machine for a published SMART model card token.

#### Identity claims

Role-based access control determines which addresses may trigger lifecycle transitions, but addresses alone do not identify the organization or role context behind an action. SMART, therefore, uses a separate identity registry in which digitally signed claims link addresses to organizational or institutional identities, with validity periods recorded on-chain. At each lifecycle transition, the current identity-claim snapshot for the actor is recorded with the corresponding ActionRecord, so later verification does not depend on the current state of the identity registry, thereby keeping lifecycle progress non-blocking while preserving accountability. For example, in our evaluations, claims issued to the developer (alice.org), reviewer (bob.org), and publisher (carol.org) accounts allowed create, review, publish, and withdrawal actions to be interpreted as role-attributed organizational actions rather than only wallet-address events. For selected transition types, the lifecycle contract can be configured to require an active identity claim before the action is accepted; panel-based quorum operations require active claims by design.

#### Operational cost

We executed the lifecycle scenarios across multiple Polygon Amoy runs and assessed operational cost over their combined transactions, for the evaluated non-admin lifecycle operations shown in Table 3. Median gas use ranged from 352 722 gas for validateModelCard, a status update that records the new lifecycle state and its audit entry without persisting new content, to 852 399 gas for reviseModelCard, which writes updated card metadata together with the immutable revision record. The gas profile reflects the documentation function of each transition rather than arbitrary network variation. Low-cost transitions primarily record a reviewed lifecycle state and its associated ActionRecord, whereas higher-cost transitions persist additional governance evidence: creation initializes a new SMART model card record, publication issues provenance through a separate contract call, and revision operations preserve updated metadata and revision history. Across the combined transactions, transaction-level CVs ranged from 1.62% to 7.24%, with the higher relative variance on smaller-magnitude operations following the inherent denominator effect of the CV metric and from per-call differences in calldata size for operations that accept variable-length string arguments (eg, metadata URI). Because gas accounting depends on contract execution (storage writes, calldata, and internal calls) rather than on the specific blockchain network, the same gas profile applies on any Ethereum-compatible blockchain on which SMART is deployed. The relayer pattern further reduces operational burden by allowing end-user externally owned accounts to perform lifecycle actions without holding POL (Polygon’s native token); gas is paid by the relayer/deployer account.

**Table 3.** Operational gas cost by SMART lifecycle operation on Polygon Amoy.

Operation	Lifecycle role	*n*	Median (gas)	Coefficient of variation (CV)
createModelCard	Initial mint	152	653 345	3.68%
submitForEvaluation	Submit for review	165	381 906	6.13%
validateModelCard	Approve at review	137	352 722	6.19%
requestRevision	Reviewer requests modifications	54	446 058	4.02%
reviseModelCard	Author revises card content	52	852 399	1.62%
rejectModelCard	Final reject	43	423 182	5.94%
publishModelCard	Publish validated card	117	752 306	3.12%
deprecateModelCard	Withdrawal/deprecation	16	423 495	7.24%

Counts (*n*) reflect successful Amoy transactions (status = 1) combined across the Amoy runs; reverted transactions are excluded. Per-operation *n* differs because each scenario invokes only the relevant subset of lifecycle calls. reviseModelCard aggregates transactions entering through both/revise and/submit-revision backend routes, which invoke the same on-chain function. reviseModelCard is the developer response to requestRevision  *within the same token record* (patch-version bump, eg, 1.0.0 → 1.0.1); successor versions across token IDs follow a separate lineage path and are not aggregated here. By construction, $n_{\mathrm{reviseModelCard}}\leq n_{\mathrm{requestRevision}}$.

## Discussion

### Scope of provision coverage

Table 4 summarizes how the in-scope provisions from the source-corpus extraction relate to SMART’s Schema, Lifecycle, and Chain components. The TransparencyInformation row indicates that 11 canonical provisions, restated across 22 source paragraphs and 8 source-corpus bearers, were attributed to the Schema component and represented in Sections 1 and 2 as identification, intended use, and clinical context. For instance, the FDA lifecycle draft guidance states that *For AI-enabled devices … manufacturers should provide labeling material … describing the instructions for use, the device*’*s indication, intended use, risks, and limitations* (p. 15).^6^ SMART represents this type of information through structured SMART model card fields rather than through a lifecycle transition. By contrast, rows such as ChangeNotification and PostMarketMonitoringReport are attributed to Lifecycle because their primary representation is a documentation action, state, or audit record. Overall, we argue that SMART can serve as an initial framework for representing clinical AI documentation as both a structured SMART model card record and a governed documentation lifecycle. It should be noted that Table 4 reports source-corpus attribution rather than regulatory conformity.

**Table 4.** Attribution of in-scope canonical provisions within SMART.

Component	Artifact	Prov.	Para.	Actors	Sources	Sec.	SMART coverage
Schema	TransparencyInformation	11	22	8	MDR, AIA, TPLC, PCCP	1, 2	Identification; intended use Clinical context
Schema	TechnicalDocumentation	11	17	6	MDR, AIA	All 7	Complete SMART card structure
Schema	ConformityAssessment	9	13	3	MDR, AIA	7	Structured documentation package
Schema	DeviceIdentifier	6	8	3	MDR, AIA	1	Device identifier metadata
Schema	PerformanceMetrics	5	5	2	MDR, TPLC, PCCP	5	Performance metrics Validation status
Schema	SeriousIncidentReport	4	4	3	MDR, AIA	7	Caveats; limitations Supporting documents
Schema	RiskManagementSystem	2	2	2	MDR, TPLC	7	Benefit-risk summary
Schema	TemplateForm	1	2	1	AIA	All 7	Structured model-card template
Schema	TrainingDataDescription	1	1	1	AIA	3	Source datasets Data provenance
Schema	IntendedUse	1	1	1	MDR	2	Intended use Clinical context
Lifecycle	ChangeNotification	7	12	3	MDR, AIA, PCCP	N/A	Review; revision Action records
Lifecycle	PostMarketMonitoringReport	4	7	4	MDR, AIA	N/A	Post-publication records Audit trail
Lifecycle	ChangeControlPlan	3	8	1	PCCP	N/A	Version lineage Change-control workflow
Schema+Lifecycle	CorrectiveAction	4	6	3	MDR, AIA	7	Caveats; withdrawal/deprecation Successor versioning
Schema+Lifecycle	PostMarketSurveillancePlan	2	2	2	MDR	7	Surveillance planning Lifecycle governance
Lifecycle+Chain	Registration	1	1	1	MDR	N/A	On-chain registration

Prov. = canonical actor, action, and artifact provisions per Pseudocode 1; Para. = source paragraph restatements; Actors = distinct regulated bearers associated with the artifact; Sec. = SMART model card section; N/A = provision is represented through lifecycle or chain functionality rather than a SMART model card section. Sources: MDR = EU MDR^3^; AIA = EU AI Act^4^; TPLC = FDA lifecycle draft^6^; PCCP = FDA PCCP.^23^

### Blockchain applicability

As described earlier, SMART’s lifecycle and audit mechanism are implemented as Solidity smart contracts and, therefore, deploy on any Ethereum-compatible blockchain. The deployment choice, for example, a local single-node setup (Hardhat for internal governance) or a public proof-of-stake network for cross-institutional settings, is determined by the adopting institution rather than fixed by the framework. We evaluated SMART on the Polygon Amoy testnet as an instance of this public-network case, where role-attributed activity becomes externally inspectable: across 748 successful transactions, each actor invoked only the operations associated with its assigned role (Figure 5), while unauthorized cross-role calls reverted under the contract access-control rules. (dave.org and eve.org were registered as additional Publisher and Reviewer accounts, respectively, for panel-based quorum scenarios; both are omitted from Figure 5 because neither had successful transactions in the captured Amoy runs.)

**Figure 5.**
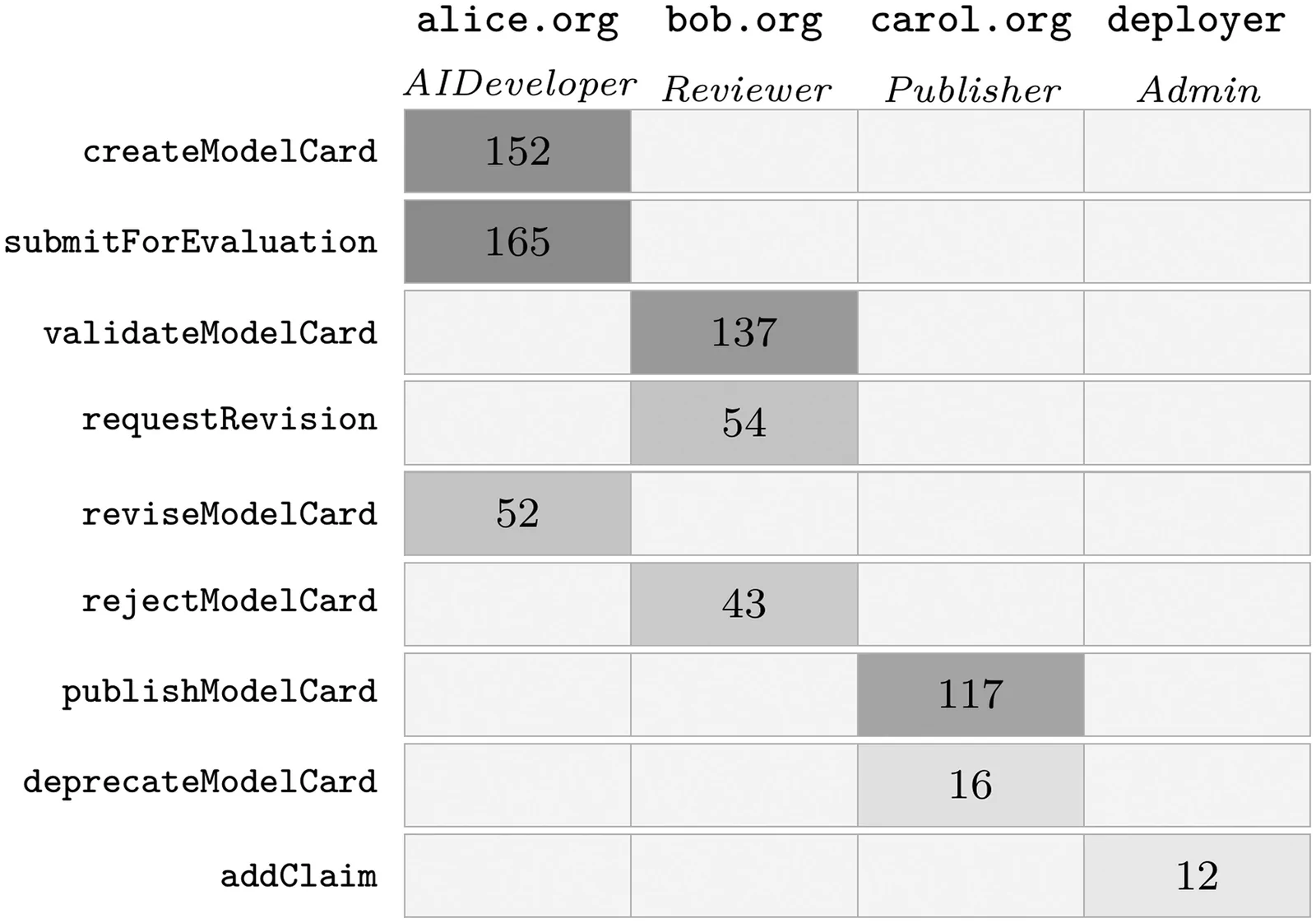
Per-actor operation distribution on Polygon Amoy (n=748 successful transactions combined across the Amoy runs; per-actor subtotals 369, 234, 133, and 12 from left to right). Cells show absolute transaction counts. Blank cells indicate operations the actor’s assigned role does not permit. The reviseModelCard count includes transactions entering through both the /revise and /submit-revision backend routes, which invoke the same on-chain contract function.

### Practical implications

One potential practical use of SMART is to support more inspectable preliminary review by stakeholders considering the use of a clinical AI system. For example, structured documentation of clinical context and intended use, together with OMOP concept sets and cohort criteria, can help assess whether a model’s development setting aligns with a deployment site’s patient population using tools such as OHDSI ATLAS. This is particularly relevant in settings where OMOP-based data harmonization is already used, including networks such as DARWIN EU^30^ and EHDEN.^31^ SMART also makes documentation history more transparently verifiable. For example, the EU MDR states that *Importers shall verify that the device is registered in the electronic system in accordance with Article 29…* (p. 27).^3^ SMART does not implement this regulatory system, but reflects a similar verification pattern at the documentation level: a relying party can check recorded documentation status rather than depend only on an operator’s claim. In SMART, this is supported through on-chain registration, content hashes, and ActionRecords, so mismatches between a retrieved model card and its recorded version become detectable through hash verification.

### Limitations

SMART is an implementable documentation framework rather than a legal interpretation or demonstration of regulatory conformity. The source-corpus extraction was used to guide the framework’s design scope, not to determine whether a specific AI system qualifies as a medical device, high-risk AI system, or compliant product. Some SMART fields, such as benefit-risk summary, surveillance-planning information, validated metrics, and lifecycle status, correspond to concepts that in practice are embedded in broader institutional, manufacturer, notified-body, or regulatory processes. In SMART, these concepts are represented as structured documentation elements and lifecycle records; the framework does not operationalize the broader approval, conformity-assessment, post-market surveillance, or clinical-validation workflows behind them. In the current implementation, validated-metric entries are documented but not independently attested at the metric level. Future work includes linking validated-metric entries to role-based attestations rather than developer-reported values alone, and connecting these documentation elements to executable review workflows, for example by linking validated-metric entries to model artifacts, evaluation datasets, and analysis scripts so that reviewers can reproduce or rerun claimed performance estimates within a controlled environment.

## Conclusions

In this article, we presented SMART, an implementable framework for structured, lifecycle-aware clinical AI documentation. A key idea in SMART was to bring together clinical context, OMOP-linked cohort description, structured performance reporting, role-attributed lifecycle governance, and verifiable documentation history within a single documentation workflow. The framework showed how intended use, population assumptions, evidence status, lifecycle state, and documentation changes can remain explicit and inspectable over time, rather than being dispersed across free-text SMART model card descriptions, local files, and operator-controlled history. More broadly, our source-corpus analysis suggests that clinical AI documentation frameworks should not be developed in isolation from regulatory, guidance, and reporting provisions, even when the resulting framework is not itself a demonstration of regulatory conformity. Overall, SMART contributes an implementable documentation framework for preliminary model assessment and traceability, not a mechanism for certifying safety, establishing clinical utility, or demonstrating regulatory conformity.

## Data Availability

The smart-model-card,^19^^,^^20^smart-omop,^21^ and the reference implementation of the SMART framework are available.^16^
